# Cardiac-specific overexpression of serum response factor regulates age-associated decline in mitochondrial function

**DOI:** 10.1007/s11357-025-01629-2

**Published:** 2025-03-31

**Authors:** Pankaj Patyal, Gohar Azhar, Xiaomin Zhang, Ambika Verma, Jeanne Y. Wei

**Affiliations:** Donald W. Reynolds Department of Geriatrics and Institute on Aging, University of Arkansas for Medical Sciences, Little Rock, AR 72205 USA

**Keywords:** Serum response factor, Mitochondria, Cardiac aging, Oxidative stress, Calcium homeostasis, MAPK

## Abstract

Cardiac aging is an intrinsic process that leads to impaired heart function, along with cellular and molecular changes. Recent research highlights the important role of mitochondria in cardiac function, due to the heart's high energy demands. Serum response factor (SRF), a transcription factor involved in regulating actin and smooth muscle gene expression, is well known as a regulator of various aspects of cardiac function. However, its role in mitochondrial regulation and cardiac aging is poorly understood. Our laboratory generated a transgenic mouse model with cardiac-specific overexpression of SRF, which exhibits characteristics of diastolic dysfunction and accelerated cardiac aging in young adult transgenic mice. In this study, we tested how cardiac-specific overexpression of SRF affects age associated mitochondrial dysfunction in the heart. Our results showed that cardiac specific SRF overexpression reduced the lifespan of mice and induced cardiomyopathy. Histological analysis revealed cardiac hypertrophy and fibrosis in transgenic mice hearts. SRF overexpression led to significant alterations in mitochondrial structure and function, including reduced mitochondrial biogenesis and dysregulation of oxidative phosphorylation. These changes were accompanied by increased oxidative stress, a decline in antioxidant enzyme activity, and disrupted calcium handling. Moreover, cardiac-specific SRF overexpression activated the MAPK signaling pathway. Our findings were further corroborated by similar mitochondrial dysfunction observed in a human cardiomyocyte cells transfected with SRF plasmid. Taken together, these findings suggest that SRF plays a novel role in cardiac aging, thus establishing SRF as a potential therapeutic target for mitigating age-associated decline in mitochondrial function and preserving cardiac health in older adults.

**Supplementary Information:**

The online version contains supplementary material available at 10.1007/s11357-025-01629-2.

## Introduction

Mitochondria are essential organelles integral to the preservation of cellular and physiological homeostasis [1, 2]. They play crucial roles in cellular bioenergetics, signaling, calcium equilibrium, cellular proliferation, and programmed cell death [3–6]. Importantly, mitochondria also play a critical role in cardiac aging, as the heart’s persistent, high demand for energy makes it especially reliant on optimal mitochondrial function [7]. However, with aging, mitochondrial efficiency decreases in cardiac cells, thus contributing to a range of age-related cardiovascular conditions, including heart failure, arrhythmias, and atherosclerosis [8, 9]. An accumulation of mitochondrial dysfunction, marked by reduced ATP synthesis, heightened oxidative stress, impaired mitochondrial dynamics (e.g., fission and fusion), and compromised mitophagy—the process by which damaged mitochondria are selectively degraded—are characteristics of cardiac aging [10–12]. These mitochondrial changes decrease myocardial contractility, disrupt calcium homeostasis, and increase susceptibility to cardiac injury [13–15]. Furthermore, the gradual accumulation of damage and mutations in mitochondrial DNA (mtDNA) exacerbates these functional impairments [16, 17]. Thus, we must understand the molecular processes that drive mitochondrial dysfunction and in turn cardiac aging.

Recent studies emphasized the critical role of transcriptional regulators in maintaining mitochondrial health during aging. One such regulator, serum response factor (SRF), a transcription factor traditionally associated with muscle differentiation and growth, has emerged as a key player in the modulation of cardiac function and remodeling [18–20]. SRF is a prominent member of the MADS-box family of transcriptional activators, known to regulate a myriad of genes critical to cellular proliferation and differentiation [21]. SRF exerts its regulatory influence by binding to the serum response element, which harbors a conserved CArG box motif (CC(A/T)GGG), a hallmark of SRF binding specificity [22]. Notably, expression of SRF increases during development and aging [23–25], and expression levels of various SRF target genes, such as atrial natriuretic factor, skeletal α- actin, cardiac α-actin, α-myosin heavy chain, and β-myosin heavy chain, change during processes like developmental maturation, cardiac hypertrophy, and cardiomyopathy [26, 27]. These findings suggest that SRF regulates genes that maintain cardiac structure and function.

SRF also plays a pivotal role in regulating the heart's response to oxidative stress [28]. Through its interactions with various signaling pathways, including MAPK, and its regulation of mitochondrial function and antioxidant genes, SRF helps to mitigate the cellular damage caused by oxidative stress [29–31]. In the context of cardiac aging and cardiovascular disease, the ability of SRF to coordinate these stress responses is crucial for maintaining cardiac health and function. While SRF's involvement in the regulation of cardiac contractility and hypertrophy is well documented, but how SRF affects mitochondrial function, particularly in the context of cardiac aging, remains underexplored.

Previously, our group has shown that increased levels of SRF in transgenic mouse line carrying human SRF under control of the Myh6 promoter resulted in cardiomyopathy and early mortality [33]. Our laboratory has developed a mouse model that recapitulates functional cardiac aging, showing clear evidence of accelerated cardiac aging in young transgenic mice. We and others have demonstrated that SRF is not only a key transcription factor involved in cytoskeletal dynamics and cellular growth but also plays a critical role in mitochondrial function and cellular energy homeostasis [34–37].

This study aims to test the hypothesis that cardiac-specific overexpression of serum response factor regulates age-associated mitochondrial dysfunction. By utilizing a transgenic mouse model with cardiac-specific SRF overexpression, we sought to uncover the molecular mechanisms through which SRF regulates mitochondrial homeostasis in the aging heart. Understanding the relationship between SRF and mitochondrial function could provide insights into novel therapeutic strategies for preserving cardiac health in the aging population.

## Materials and methods

### Animals

Mice were obtained from transgenic colonies that overexpress SRF (SRF-Tg), established on the FVB/N genetic background as previously described [32]. The SRF transgene contains an α-MHC promoter, which drives the expression of full-length SRF cDNA, leading to cardiac-specific elevation of SRF protein, resulting in the characteristic cardiac phenotype of this mouse model [33]. The transgenic line was maintained in the hemizygous state to generate non-transgenic littermate controls (non-Tg).

Mice were housed in the Division of Laboratory Animal Medicine facility at the University of Arkansas for Medical Sciences, in ventilated cage racks within a controlled environment (22 °C, 30–50% humidity) on a 14-h light/10-h dark cycle, was fed ad libitum with (irradiated) food and water (treated with a reverse osmosis system). Each breeding cage housed 1 male and 2 female breeders; typically, only the male mouse carried the transgene. SRF-Tg mice were identified through PCR analysis of genomic DNA extracted from tail biopsies, in conjunction with the primer pair as described previously [32].

Experimental groups contained both male and female mice at ages of 2-, 3-, 6- and 24- months old. Experiments involving mice were approved by the Institutional Animal Care and Use Committee at the University of Arkansas for Medical Sciences, in accordance with the Public Health Service Policy on Humane Care and Use of Laboratory Animals, the National Research Council's Guide for the Care and Use of Laboratory Animals, and the ARRIVE guidelines [38].

### Mouse survival study

All SRF-Tg and non-Tg littermates (FVB/N background) were maintained on a 14-h light/10-h dark cycle. Mice were housed in groups of 3 to 5 same‐sex littermates under pathogen‐free conditions. Mice were monitored twice a week and weighed monthly but were otherwise left undisturbed until they survived. Survival was assessed in both male and female mice, and all animals were dead by the time of this report. Birth and death dates of these mice were used to construct Kaplan–Meier survival curves, differences between groups were evaluated using the logrank test.

### Heart weight and body weight

The body weights of the SRF-Tg and non-Tg littermates were measured before euthanasia; littermates were weighed and euthanized at 3 months and 6 months of age. Mice were anesthetized deeply with carbon dioxide and euthanized by cervical dislocation. After euthanasia, heart tissues were dissected and weighed. The ratio of heart weight to body weight was calculated as heart weight (mg)/body weight (g).

### Histological analysis

SRF-Tg mice and non-Tg littermates were anesthetized with carbon dioxide and euthanized via cervical dislocation. After euthanasia, hearts were excised and placed in a 25 mM KCl solution to induce diastolic arrest. Following a brief PBS rinse, hearts were immediately fixed in 10% neutral-buffered formalin at room temperature for 24 h. Tissue processing and imaging were performed by the Experimental Pathology Core at University of Arkansas for Medical Sciences. Briefly, the atrial segment was separated from the ventricular segment, and ventricular tissue slices of 3- to 4-mm thickness were cut, embedded in paraffin and sectioned at a thickness of 5 µm. Sections were deparaffinized in xylene, rehydrated in ethanol, and prepared for histological analysis. Staining with hematoxylin and eosin and Masson’s trichrome (Poly Scientific; Bayshore, NY, USA) was performed according to the manufacturer’s instructions. Images of SRF Tg with age and gender matched, non-Tg hearts were taken with a Leica (Aperio) Scanscope CS2. Cardiomyocyte and fibrotic cross-sectional areas were quantified from each heart cross-section in all groups via ImageJ software (version 1.54g, NIH, USA). Ten different regions were randomly selected from the left ventricular free wall and from each image at 20 × magnification and cross-sectional area was measured from 50 or more distinct cardiomyocytes. The borders of individual cardiomyocyte were meticulously traced, and pixel counts within each boundary were calibrated to a scale for precise area measurement. The mean ± SD area measurements of cardiomyocytes were obtained as representative data. Fibrotic areas were quantified using the Masson Trichome-stained cross-sections of hearts at the level of the left ventricular wall papillary muscle and averaged to obtain representative data. The fibrotic area was measured as blue stained-positive area normalized to the high-power field of total myocardial area.

### Transmission electron microscopy

Mice were anesthetized with carbon dioxide and euthanized by cervical dislocation. Mouse hearts were processed as described above and fixed with freshly made 3% glutaraldehyde in 1 × PBS and stored in the same solution at 4 °C until transmission electron microscopy was performed at the Digital Microscopy Core at University of Arkansas for Medical Sciences. The detailed method was described previously [39]. Briefly, small sections of the wall of the left ventricle (LV) were washed in 1 M phosphate buffer (pH 7.3), postfixed in 1% osmium tetroxide for 1 h, and dehydrated through a series of graded alcohol baths. Ultra-thin Sects. (70–80 nm) were cut from blocks with a Leica EM UC7 ultra microtome. Sections were mounted on 100 mesh copper grids and imaged on a FEI Tecnai F20 200 keV Transmission Electron Microscope. To determine the mitochondrial quality, ImageJ software (version 1.54g, NIH, USA) was used to quantify and measure the different parameters. Cristae/mitochondrial area (the average percentage of cristae in the total mitochondrial area of different EM sections), and mitochondrial damage (the average percentage of damaged mitochondria to the total image area).

## Western blot analysis

Heart tissue from the LV was homogenized in RIPA lysis buffer (Santa Cruz Biotechnology, Dallas, Texas, USA, cat. no. sc-24948A), followed by centrifugation at 12,000 rpm for 20 min at 4 °C. Lysate protein concentrations were determined with the Pierce BCA Protein Assay Kit (ThermoFisher Scientific, Waltham, MA, USA, cat. no. 23227). Total protein (10 µg) was loaded on sodium dodecyl sulfate–polyacrylamide gel electrophoresis gels and were electroblotted onto nitrocellulose membrane. Membranes were blocked with 5% nonfat dry milk in Tris-buffered saline with Tween-20 (TBST) for 1 h at room temperature, washed 3 times with TBST (5 min each), and incubated overnight at 4 °C with primary antibodies.

The following primary antibodies were purchased from Santa Cruz Biotechnology, Dallas, Texas, USA: SRF (1:1000, cat. no. sc-335), BNP (1:1000, cat. no. sc-271185), PGC-1α (1:1000, cat. no. sc-518025), PGC-1β (1:1000, cat. no. sc-373771), OPA1 (1:1000, cat. no. sc-393296), DRP1 (1:1000, cat. no. sc-271583), glutathione reductase (1:1000, cat. no. sc-133245), SOD-2 (1:1000, cat. no. sc-30080), SERCA2 (1:1000, cat. no. sc-376235), MEK (1:1000, cat. no. sc-81504), p-MEK (1:1000, cat. no. sc-81503), ERK1/2 (1:1000, cat. no. sc-135900), p-ERK (1:1000, cat. no. sc-136521), JNK1/2 (1:1000, cat. no. sc-7345), p-JNK (1:1000, cat. no. sc-6254), P38 (1:1000, cat. no. sc-271120), p-P38 (1:1000, cat. no. sc-7973), and GAPDH (1:1000, cat. no. sc-365062). PGC-1α (1:1000, cat. no. ab1068144), HNE (1:1000, cat. no. ab46545), and RYR2 (1:500, cat. no. ab302716) were purchased from Abcam, Waltham, MA, USA. The OXPHOS cocktail antibody (1:5000, cat. no. 45–8099) was purchased from ThermoFisher Scientific, Waltham, MA, USA.

The next day, the membrane was washed 3 times in TBST (5 min each) and incubated for 1 h at room temperature with a secondary antibody diluted 1:5000 in blocking solution. The secondary antibodies included anti-mouse HRP (Invitrogen, Carlsbad, CA, USA, cat. no. 62–6520), anti-goat HRP (Santa Cruz, Dallas, Texas, USA, cat. no. sc-20200), and anti-rabbit AP (Bio-Rad, Hercules, CA, USA, cat. no. 64251130). Immunoreactive bands were visualized with the SuperSignal West Dura Luminol/Enhancer Solution (ThermoFisher Scientific/Pierce Biotechnology, cat. no.1856145). Images were taken with iBright CL1500 (Invitrogen, Waltham, MA, USA). Densitometric analysis was conducted with ImageJ software (Version 1.54g, NIH).

### High-resolution respiratory analysis

The Oxygraph-O2k high-resolution respirometer (Oroboros Instruments, Innsbruck, Austria) was used to examine the activity of individual respiratory chain complexes (I–IV) of mitochondria. The heart tissue from the LV was excised and cut into small samples of 5 mg and put into a falcon tube with 10 mL of ice-cold BIOPS buffer. BIOPS, the relaxing and biopsy preservation solution, contains 10 mM Ca-EGTA buffer, 0.1 µM free calcium, 20 mM imidazole, 20 mM taurine, 50 mM K-MES, 0.5 mM DTT, 6.56 mM MgCl_2_, 5.77 mM ATP, and 15 mM phosphocreatine, with whole solution pH adjusted to 7. Tissue was then placed in ice-cold BIOPS in a small petri dish on ice. The tissue was minced vertically and horizontally with a sharp blade. After mincing, tissue was transferred quickly into 2 mL of ice-cold BIOPS, containing 20 µL of saponin stock solution (5 mg/mL, final concentration 50 µg/mL) for tissue permeabilization. Tissue was incubated in saponin solution on a shaker for 30 min, followed by incubation in MiR05 buffer (Oroboros Instruments, Innsbruck, Austria) for 10 min. Equal amounts (5 mg) of heart tissue from the LV of SRF-Tg and non-Tg mice were then added into respective chambers of the O2k respirometer. The detailed method of substrate-inhibitor-titration protocol was described previously [36]. Data were exported and analyzed with DatLab 6.2 software (Oroboros Instruments, Innsbruck, Austria), and cellular respiration of each mitochondrial complex was expressed as oxygen flux [pmol/(s*mg)].

### Mouse mtDNA quantification

Genomic DNA was isolated from SRF-Tg and littermates non-Tg controls and subjected to quantify the absolute copy numbers of mouse mtDNA. Absolute Mouse Mitochondrial DNA Copy Number Quantification qPCR Assay kit (cat. no. M8948, ScienCell, Carlsbad, CA, USA) was utilized as per manufacturer’s instructions. The mouse mtDNA primer sets provided in the kit recognize and amplify a conserved region of mouse mtDNA. The single copy reference primer set recognizes and amplifies a 100 bp-long region on mouse chromosome 10 and serves as reference for data normalization. The reference genomic DNA sample with known mtDNA copy number serves as a reference for calculating the mtDNA copy number of target samples.

### Cell culture and transfection

The AC16 human cardiomyocyte cell line was purchased from American Type Culture Collection [ATCC], Manassas, VA, USA (cat. no. CRL-3568). These cells were cultured in Dulbecco’s Modified Eagle Medium-F12 medium (ATCC, Manassas, VA, USA, cat. no. 11965092). The cell culture conditions were previously described in detail [40]. SRF plasmid and empty vector used in this study have been previously described [41]. SRF plasmid DNA transfections were performed with Lipofectamine 2000 (Invitrogen, Carlsbad, CA, USA) according to the manufacturer’s instructions. The detailed protocol of transfection has been previously provided [42].

### Extracellular flux assays

Assays to determine the oxidative consumption rate (OCR), extracellular acidification rate, and ATP rate were performed with the Seahorse XFe96 Analyzer (Agilent, Santa Clara, CA, USA). Equal numbers of AC16 cells (10,000 cells) were seeded per well in an XF Cell Culture Microplate (Agilent, Santa Clara, CA, USA) with complete medium at 37 °C, 5% CO_2_, and 100% humidity. The cells were transfected with empty vector or plasmid containing SRF for 24 h. After 24 h of transfection, the cells were washed with Seahorse XF DMEM medium (pH 7.4) and left in Seahorse XF DMEM medium for 1 h at 37 °C in a non-CO_2_ incubator. The real-time cell metabolic function was measured with the different test kits as explained previously [43, 44]. The data from OCR, glycolytic rate, and ATP assay experiments were normalized to an equal number of cells in both transfected conditions. After each run, a Seahorse plate was used to perform cell count via the trypan blue exclusion method; data were normalized accordingly.

### Statistical analysis

Experiments were performed at least 3–5 times, with n denoting the number of experiments unless otherwise indicated, and the data are presented as means ± SD. Differences between groups were analyzed with one-way or two-way analysis of variance (ANOVA) followed by a Bonferroni multiple comparisons test or suitable multiple comparisons test. Results with *P*-values < 0.05 were considered statistically significant. Statistical analyses were performed with Prism 10.0 (GraphPad Software, San Diego, CA, USA).

## Results

### Overexpression of SRF in the mouse heart reduces lifespan and induces cardiomyopathy

**Fig. 1:**
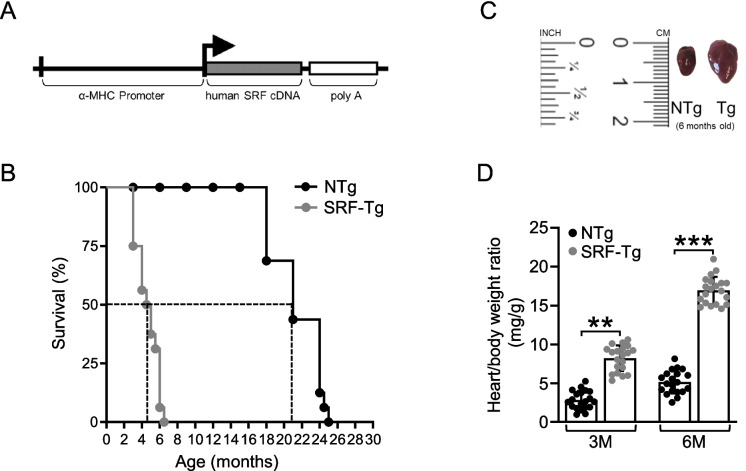
Overexpression of *SRF* reduces lifespan and induces cardiomyopathy. **A** DNA construct for the generation of *SRF* transgenic mice. *SRF*, the full-length cDNA of human *SRF* gene; *α-MHC*, α-myosin heavy chain; Hgh, the polyA sequence of human growth hormone gene. **B** Kaplan–Meier graph of the maximum lifespan in SRF-Tg mice (gray, *n* = 20) compared with non-Tg littermate mice (black, *n* = 20). The differences between groups were evaluated using the log-rank test. **C** Hearts of SRF-Tg and non-Tg mice at 6 months of age. The ruler shows inches and centimeters markings. **D** Ratio of heart weight to body weight (mg/g) in non-Tg (NTg) and SRF-Tg mice at 3 months and 6 months of age. All data are presented as means ± SD, *n* = 20, where n represents biological replicates. **P < .005, ***P < .001, using two-way ANOVA with Bonferroni multiple comparisons test

Previously, we generated a transgenic mouse model to examine the effects of increased SRF expression in the heart [32]. The transgenic construct contains human SRF cDNA under the control of the *α-MHC* promoter and the polyA tail of the human growth hormone gene (Fig. 1A). Multiple founder transgenic mice were generated with different copy numbers, but only one founder mouse which has one copy number of the *SRF* transgene, successfully reproduced heterozygous transgenic progeny [33].

We first evaluated how cardiac-specific overexpression of SRF affects survival. To do so, we followed SRF-Tg and non-Tg littermate control mice throughout their entire lifespan. As illustrated in our Kaplan–Meier survival curve, SRF-Tg mice only survived for 6–7 months and had a significantly shorter mean lifespan than their littermate controls, significantly different (*p* < 0.001) (Fig. 1B). In addition, at 6 months of age, SRF-Tg mice showed cardiomyopathy with increased heart size, increased heart weight, and four-chamber dilatation (Fig. 1C). Furthermore, at both 3 months and 6 months of age, SRF-Tg mice had a significantly higher ratio of heart weight to body weight (mg/g) than non-Tg littermates, with significantly different at (*p* < 0.01, 3 months) and (*p* < 0.001, 6 months) (Fig. 1D). The phenotypical examination of these transgenic mice with cardiomyopathy at or near death revealed rapid breathing and limp body with minimal or no reflex responses (Supplementary Fig. 1). Taken together, these findings indicate that SRF plays a critical role in regulating cardiac health and that our model can be used to study heart disease mechanisms.

### Overexpression of SRF in the mouse heart induces hypertrophy and fibrosis

**Fig. 2:**
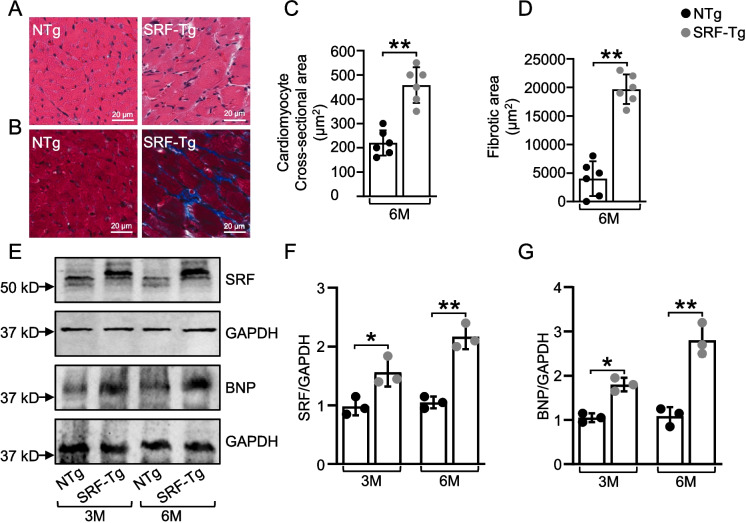
Histological and western blot analysis of cardiac hypertrophy and fibrosis. **A** Left ventricular cardiac myocytes of SRF-Tg and non-Tg mice at 6 months of age stained with hematoxylin and eosin stain. **B** Masson trichrome staining of the left ventricle section of SRF-Tg and non-Tg mice at 6 months of age. Collagen stains blue with trichrome. **C** Quantitative analysis of cell surface cross-sectional area of cardiomyocytes. Values are mean ± SD (*n* = 6). **D** Quantitative analysis of fibrosis area. The fibrotic area was measured as blue stained-positive area normalized to the high-power field of total myocardial area. Values are mean ± SD (*n* = 6). **E** Representative western blot images of BNP, a marker for left ventricle hypertrophy and total SRF expression at 3 months and 6 months of age. GAPDH was used as a loading control. **F**, **G** Quantification of relative protein levels normalized against GAPDH. Data are mean ± SD (*n* = 3). N represents independent biological repeats. A 20 × objective is used; scale bars indicate 20 μm. *P < .05, **P < .01, using two-way ANOVA with Bonferroni multiple comparisons test

Hematoxylin and eosin and Masson's trichrome-stained sections of 6-month-old mice exhibited a phenotype of hypertrophy and cardiac fibrosis (Fig. 2). Histological examination showed increased cell size and large nuclei in the cardiomyocytes of SRF-Tg mice, thereby confirming cardiac hypertrophy (Fig. 2A). The cardiomyocytes area was significantly higher (*p* < 0.05) in SRF-Tg mice (Fig. 2C). Segmented images of presented H&E staining sections with marked edges to measure the cardiomyocytes area, for non-Tg and SRF-Tg mouse heart sections are shown in Supplementary Fig. 2. Masson's trichrome-stained heart sections revealed diffuse interstitial fibrosis in SRF-Tg mice (Fig. 2B) and there was significant increase (*p* < 0.05) in fold amount of fibrotic area in SRF-Tg mice (Fig. 2D).

Consistent with histological analyses, cardiac-specific overexpression of SRF caused significant upregulation of the LV cardiac hypertrophic marker BNP. Western blot analysis of SRF and BNP in 3-month-old and 6-month-old mice revealed a pronounced and sustained upregulation of both SRF and BNP as the mice aged, with significantly different at *p* < 0.05 (3 months) and *p* < 0.01 (6 months) for both (Fig. 2E–G). We also examined younger SRF-Tg mice to determine the age at which transgene expression in the heart becomes more pronounced compared to non-Tg littermates. At two months of age, SRF-Tg mice showed no changes in SRF expression levels in mice hearts compared to non-Tg mouse hearts (Supplementary Fig. 3). This highlights the temporal progression of SRF overexpression in our model. SRF overexpression may not immediately lead to a detectable increase in expression at 2 months due to a delayed response in the activation of SRF-dependent transcriptional programs in the pre-pubertal phase. Overall, our results revealed that cardiac-specific SRF overexpression exacerbated pathological cardiac hypertrophy and fibrosis.

### Overexpression of SRF in the mouse heart induces significant alterations in mitochondrial structure and modulates mitochondrial functional dynamics

**Fig. 3:**
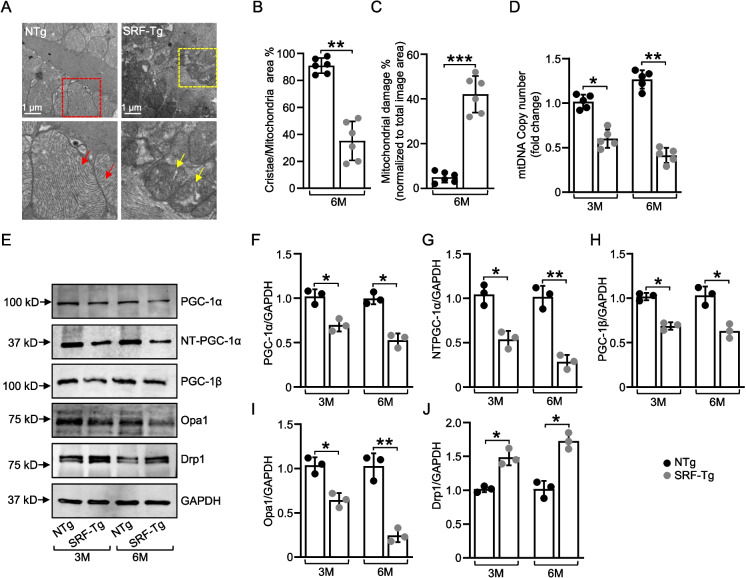
Transmission electron microscopy and western blot analysis of proteins involved in mitochondrial function and dynamics. **A** Detailed transmission electron microscopy of left ventricular tissue taken from SRF-Tg and non-Tg mice at 6 months of age. The red box in non-Tg section was magnified below; red arrows show regular arrangement of myofibrils and well-packed, regular cristae in the mitochondria. The magnified section of the yellow box in SRF-Tg below shows yellow arrows pointing toward shortened and disarrayed myofibrils and arrows indicating damaged mitochondria with loss of cristae. Scale bars indicate 1 μm (**B**) The average percentage of cristae in total mitochondrial area of different EM sections at 6 months of age (*n* = 6). **C** Percentage of damaged mitochondria of different EM sections at 6 months of age (*n* = 6). **D** Fold amount of mtDNA copy number at 3 months and 6 months of age (*n* = 5). **E** Representative immunoblots of PGC-1α, NT-PGC-1α, PGC-1β, Opa1, and Drp1 in SRF-Tg and non-Tg mice hearts at 3 months and 6 months of age. (**F, G, H, I, J**) Quantification of the relative changes in protein levels normalized against GAPDH. Data are mean ± SD (*n* = 3). N represents independent biological repeats. *P < .05, **P < .01, *** *P* < .001, using two-way ANOVA with Bonferroni multiple comparisons test

We next aimed to determine how cardiac-specific overexpression of SRF affects the ultrastructure of mitochondria in the mouse heart. Dense cristae and appropriate mitochondrial length are the structural basis for normal mitochondrial function; for this reason, we performed transmission electron microscopy to assess the mitochondrial cristae structure and overall health of mitochondria. In hearts from SRF-Tg mice, mitochondrial structures were damaged (Fig. 3A). Further, ultrastructural analysis of hearts from SRF-Tg mice revealed shortened, disarrayed myofibrils, frequently fragmented cristae, and a loss of cristae. The cristae number was significantly reduced (*p* < 0.01) in hearts from SRF-Tg mice compared with hearts from non-Tg mice; this was accompanied by overall mitochondrial damage (*p* < 0.001) (Fig. 3B, C).

We then assessed the mtDNA copy number and found that at 3 and 6 months of age, hearts of SRF-Tg mice had reduced mtDNA copy number, indicating fewer copies of mtDNA present within the mitochondria of a cell, with significantly different at *p* < 0.05 (3 months) and *p* < 0.01 (6 months). This reduction can lead to decreased energy production and impaired cellular function (Fig. 3D).

Next, we evaluated whether SRF overexpression in the heart led to a reduction in expression of transcriptional coregulators, which are involved in mitochondrial biogenesis and energetic metabolism. The expression levels of PGC-1α, *p* < 0.05 (3 months) and *p* < 0.05 (6 months), its splice variant NT-PGC-1α, *p* < 0.05 (3 months) and *p* < 0.01 (6 months), and PGC-1β, *p* < 0.05 (3 months) and *p* < 0.05 (6 months), were greatly reduced in hearts of SRF-Tg mice as compared to non-Tg mice hearts (Fig. 3E–H). SRF overexpression also significantly decreased the expression of OPA1,* p* < 0.05 (3 months) and *p* < 0.01 (6 months), which regulates mitochondrial fusion and cristae structure. In contrast, SRF overexpression increased the expression of Drp1,* p* < 0.05 (3 months) and *p* < 0.05 (6 months), a fission protein, which indicates mitochondrial fragmentation (Fig. 3E, I, and J). Taken together, our data suggest that cardiac-specific overexpression of SRF caused alterations in mitochondrial structure and modulated mitochondrial function by controlling the expression levels of proteins involved in mitochondrial biogenesis and dynamics.

### Overexpression of SRF in the mouse heart perturbs regulation of oxidative phosphorylation

**Fig. 4:**
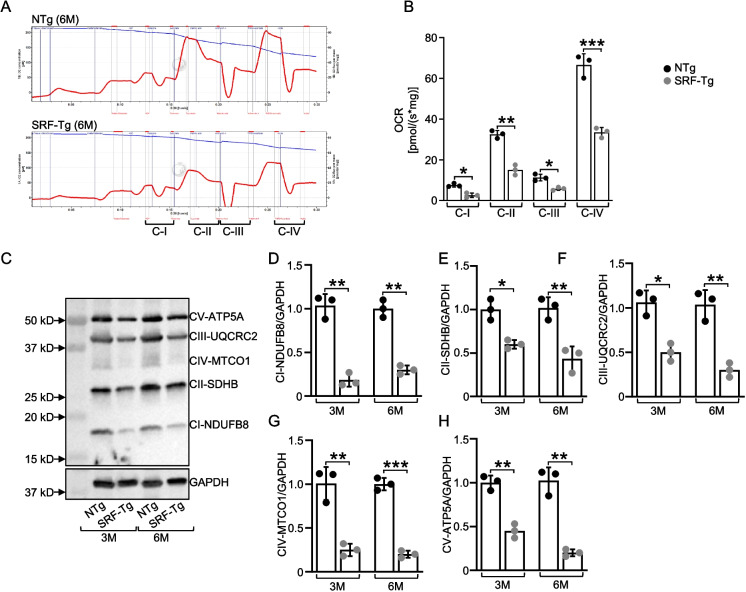
Functional analysis of mitochondrial respiratory chain. **A** Representative trace of high-resolution respirometry with a multiple substrate-inhibitor titration protocol in mice hearts of SRF-Tg comparing to non-Tg littermates at 6 months of age. Oxygen consumption is represented as a function of time. Blue lines indicate times of titrations of substrates and inhibitors. The protocol includes the following steps: malate/glutamate (Complex I-linked substrates), ADP (OXPHOS capacity), rotenone (inhibition of Complex I), succinate (Complex II), antimycin A (inhibition of Complex III), ascorbate/tetramethyl-p-phenylenediamine (Complex IV), and sodium azide (inhibition of Complex IV). **B** Quantification of complexes activity (*n* = 3). **C** Representative western blot of OXPHOS mitochondrial complexes (I–V). Antibody cocktail against complexes was used to examine the expression of mitochondrial proteins in SRF-Tg mouse hearts at 3 and 6 months of age. **D**, **E**, **F**, **G**, **H** Relative quantification of protein levels normalized against GAPDH. Data are mean ± SD (*n* = 3). N represents independent biological repeats. *P < .05, **P < .01, ***P < .001, using two-way ANOVA with Bonferroni multiple comparisons test

Next, we investigated how SRF overexpression in the mouse heart affects oxidative phosphorylation, a key process that takes place within the electron transport chain. This chain consists of a series of protein complexes (Complexes I–IV) embedded in the inner mitochondrial membrane, which facilitate electron transfer and proton pumping to generate a proton gradient used to produce ATP via ATP synthase [45]. SRF overexpression have a substrate-inhibitor downregulation in the respiratory capacity as exhibited by the high-resolution in respiratory parameters of the electron transport system. The activity of Complexes I (*p* < 0.05), II (*p* < 0.01), III (*p* < 0.05), and IV ( *p* < 0.001) was reduced significantly in the SRF-Tg mouse heart, (Fig. 4A). The integrity of the mitochondrial membrane was evaluated by including cytochrome c. The quantitative examinations of oxygen respiration rate in different complexes are shown in Fig. 4B. These findings are supported by the OXPHOS (I–V) western blot analysis data (Fig. 4C), which shows that SRF overexpression resulted in significant downregulation of key component proteins of the mitochondrial complexes of electron transport chain (Fig. 4D–H). The significant *p* values were CI- NDUFB8, *p* < 0.01 (3 months) and *p* < 0.01 (6 months), CII-SDHB, *p* < 0.05 (3 months) and *p* < 0.01 (6 months), CIII-UQCRC2, *p* < 0.05 (3 months) and *p* < 0.01 (6 months), CIV-MTCO1, *p* < 0.01 (3 months) and *p* < 0.001 (6 months), and CV-ATP5A, ( *p* < 0.01 (3 months) and *p* < 0.01 (6 months). Therefore, our data suggest that the abnormal upregulation of SRF within the myocardium disturbs the delicate balance of electron transport chain function, potentially impairing the efficiency of ATP production via oxidative phosphorylation.

### Overexpression of SRF in the mouse heart induces oxidative stress and impairs calcium signaling via reduction in SERCA2 and RyR2 expression

**Fig. 5:**
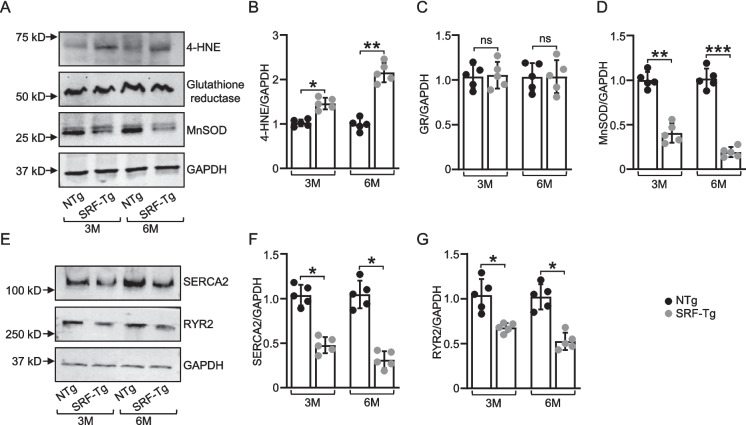
Western blot analysis of proteins involved in oxidative stress and calcium handling. **A** Representative western blot images of 4-HNE, a marker for oxidative stress, glutathione reductase, and MnSOD, an antioxidant enzyme, in SRF-Tg and non-Tg mouse hearts at 3 and 6 months of age. **B**, **C**, **D** Relative quantification of protein levels of 4-HNE, glutathione reductase, and MnSOD normalized against GAPDH in 3 months and 6 months of age. **E** Representative western blot images of SERCA2, which pumps calcium back into the sarcoplasmic reticulum, and RYR2, a calcium release channel, in SRF-Tg and non-Tg mouse hearts at 3 months and 6 months of age. **F**, **G** Relative quantification of protein levels of SERCA2 and RYR2 normalized against GAPDH. Data are mean ± SD (*n* = 5), where n represents independent biological repeats. *P < .05; **P < .01; ***P < .001; ns, *P* > .05, using two-way ANOVA with Bonferroni multiple comparisons test

In the context of altered mitochondrial function, where SRF-induced changes in gene expression disrupt normal oxidative phosphorylation, we asked whether this disruption led to the onset of oxidative stress. Our western blot analysis of 4-HNE, a biomarker for oxidative stress, showed that at 3 months (*p* < 0.05) and 6 months (*p* < 0.01), SRF-Tg mice had more 4-HNE in their hearts than non-Tg mice (Fig. 5A and B). This increase in 4-HNE protein indicates heightened oxidative stress in the cardiac tissue of these mice at both age points.

We then examined the expression levels of antioxidant enzymes. Although oxidative stress increased in the SRF-Tg heart, expression of MnSOD decreased significantly with *p* < 0.01 at 3 months and *p* < 0.001 at 6 months of age (Fig. 5A and D). This reduction in MnSOD expression suggests a compromised antioxidant defense system, which may contribute to the elevated oxidative stress observed in the cardiac tissue. We did not observe any change in expression of glutathione reductase (Fig. 5A and C), indicating that despite the increase in oxidative stress and changes in other antioxidant enzymes, the activity of glutathione reductase remained unaffected in the mouse heart tissue. Furthermore, expression of SERCA2 and RyR2, which are crucial for calcium handling and myocardial contractility, was decreased (Fig. 5E–G). They both were significantly decreased as compared to the non-Tg littermates with *p* < 0.05 at 3 months and *p* < 0.05 at 6 months of age. This reduction, coupled with increased oxidative stress, suggests that oxidative stress might contribute to impaired calcium handling in the cardiac cells, potentially leading to disrupted myocardial function and contractility.

### Overexpression of SRF in the mouse heart induces MAPK activation

**Fig. 6:**
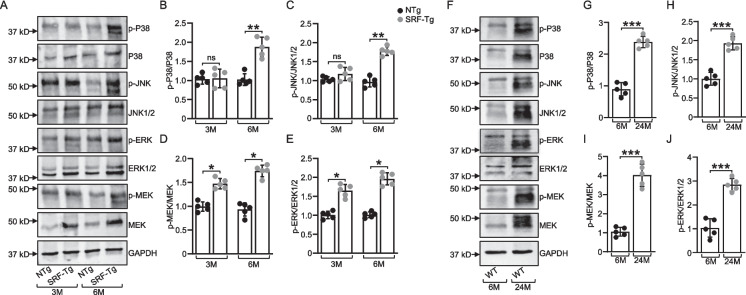
Overexpression of *SRF* activates the MAPK signaling pathway. **A** Representative immunoblots of total and phosphorylation of MEK, ERK1/2, JNK1/2, p38 MAPK in SRF-Tg and non-Tg mouse hearts at 3 months and 6 months of age. **B**, **C**, **D**, **E** Quantification of the relative changes in phosphorylation of MEK, ERK1/2, JNK1/2, and p38 from total protein. **F** Representative immunoblots of total and phosphorylated MEK, ERK1/2, JNK1/2, p38 in young (6 month) versus old (24 months) Wild-type mice hearts. **G**, **H**, **I**, **J** Quantification of the relative changes in phosphorylation of MEK, ERK1/2, JNK1/2, and p38 from total protein expression. Molecular weight (kDa) are indicated. GAPDH was used as a loading control. Data are expressed as mean ± SD (*n* = 5), where n represents independent biological repeats. *P < .05; **P < .01; ***P < .001; ns, *P* > .05, using two-way ANOVA with Bonferroni multiple comparisons test

To determine whether SRF overexpression activates cardioprotective pathways, such as the MAPK cascade, we assessed the phosphorylation and activation states of MEK, ERK1/2, JNK1/2, and p38 MAPK. Our data indicate that in the SRF-Tg mouse heart at both 3 months and 6 months, p38 MAPK was activated and at 6 months of age, p38 was phosphorylated, with significant increase with *p* < 0.01 at 6 months of age, highlighting sustained activation of this critical stress-responsive kinase (Fig. 6A and B).

Further, we found that JNK (with *p* < 0.01 at 6 months of age), ERK (with *p* < 0.05 at both 3 and 6 months of age) and MEK (with *p* < 0.05 at both 3 and 6 months of age) were activated and phosphorylated in the SRF-Tg mouse heart at both 3 months and 6 months, suggesting persistent activation of the MAPK signaling pathway in these mice (Fig. 6A, C, D, and E). This persistent phosphorylation suggests an ongoing activation of these kinases in the MAPK pathway, which may play a role in mediating cellular responses to stress and contributing to the long-term effects of SRF overexpression in the heart.

We then examined and compared the hearts of young and old wild-type mice for MAPK activation to assess potential age-related differences in the signaling pathways. Our data indicate that all these kinases—MEK (with *p* < 0.001), ERK1/2 (with *p* < 0.001), JNK1/2 (with *p* < 0.01), and p38 MAPK (with *p* < 0.001)—were significantly activated in the 24-month-old mouse hearts compared with the 6-month-old mouse hearts (Fig. 6F–J). This increased activation suggests an age-dependent upregulation of the MAPK signaling pathways, which may reflect enhanced cellular stress or adaptive responses to aging. Additionally, the activation of MAPKs in the aging heart is consistent with activation of MAPKs in the SRF-Tg mouse heart, as SRF expression is upregulated during cardiac aging. This suggests that the elevated SRF levels in the aging heart may contribute to the enhanced activation of MAPK pathways observed in older mice, potentially linking SRF overexpression to age-related changes in cardiac signaling.

### Overexpression of SRF in a human cardiomyocyte cell line results in mitochondrial dysfunction

**Fig. 7:**
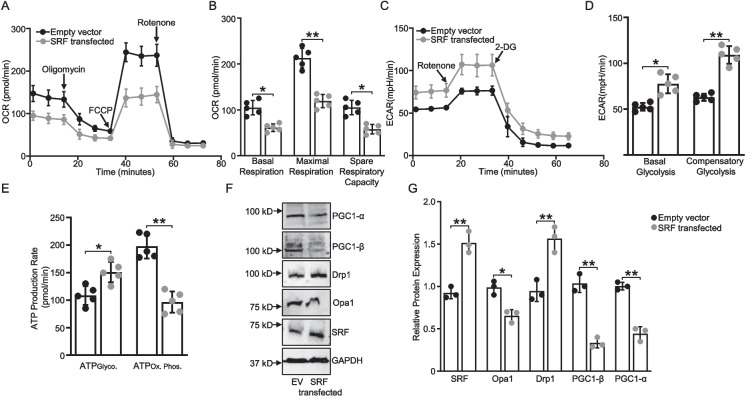
Overexpression of *SRF* alters mitochondrial bioenergetics. AC16 cells were transfected with either an *SRF* plasmid or an empty vector. Various mitochondrial assays were performed. **A** Seahorse XF Cell Mito Stress Test results of cells transfected with either empty vector or *SRF* vector shown as mean ± SD normalized to equal number of cells. Oligomycin was injected to inhibit ATP-linked mitochondrial respiration as a measure of mitochondrial ATP production. To determine maximal respiration, the mitochondrial uncoupler FCCP was injected. To determine the non-mitochondrial respiration to the oxygen consumption rate, rotenone was injected, inhibiting mitochondrial respiration. **B** Quantification of basal respiration, maximal respiration, and spare respiratory capacity calculated from Seahorse XF Cell Mito Stress Test (*n* = 5). **C** Glycolytic rate assay shown as mean ± SD normalized to equal number of cells. Rotenone/Antimycin A mix was injected to block mitochondrial activity and 2-DG (to inhibit glycolysis) as an internal control. **D** Graphs show the calculated glycolytic parameters of basal and compensatory glycolysis (*n* = 5). **E** Real-time ATP rate assay in AC16 cells (*n* = 5). **F** Representative immunoblots of PGC-1α, NT-PGC-1α, PGC-1β, Opa1, and Drp1 in *SRF*-transfected and empty vector-transfected AC16 cells. **G** Quantification of the relative changes in protein levels normalized against GAPDH. Data are mean ± SD (*n* = 3). N represents independent biological repeats. *P < .05, **P < .01, using two-way ANOVA with Bonferroni multiple comparisons test

To better understand the compensatory mechanisms involved in mitochondrial function, including oxidative phosphorylation, glycolysis, and total ATP production with SRF overexpression, and to further validate our findings, we used human cardiomyocyte cell line AC16. We transfected the AC16 cells with an SRF plasmid and assessed mitochondrial function. Our data indicate that SRF overexpression via transfection in AC16 cells reduced oxidative phosphorylation and increased glycolysis, likely to compensate for the decrease in oxidative phosphorylation (Fig. 7A and C). Quantification of OCR revealed that following transfection, AC16 cells had significant reduced basal (*p* < 0.05) and maximal respiration (*p* < 0.01) along with decreased spare respiratory capacity (*p* < 0.05) (Fig. 7B). In compensatory mechanism, both basal (*p* < 0.05) and compensatory glycolysis (*p* < 0.01) were significantly increased in SRF-transfected AC16 cells (Fig. 7D). Furthermore, total ATP production was reduced (*p* < 0.05) in SRF-transfected AC16 cells, which indicates a shift in cellular energy metabolism, likely due to impaired oxidative phosphorylation and increased reliance on glycolysis for ATP generation (Fig. 7E).

These findings were further corroborated by a significant reduction in key transcriptional regulators of mitochondrial function, including PGC-1α (*p* < 0.01) and PGC-1β (*p* < 0.01), which are essential for the maintenance of mitochondrial biogenesis and activity. SRF-transfected cells exhibited a reduction in OPA1 levels (*p* < 0.05), coupled with an increase in DRP1 expression (*p* < 0.01), suggesting a shift toward mitochondrial fission and potential disruption of mitochondrial dynamics. SRF transfection was confirmed via western blot, which showed increased expression of SRF (*p* < 0.01) in AC16 cells following transfection (Fig. 7F and G).

In summary, SRF transfection in AC16 cells impaired oxidative phosphorylation, shifted energy metabolism toward glycolysis, reduced ATP production, and disrupted mitochondrial dynamics. These findings are consistent with data from the SRF-Tg mouse model, highlighting SRF's role in regulating mitochondrial function and maintaining cellular energy homeostasis.

## Discussion

Our study investigated the effects of cardiac-specific overexpression of SRF in transgenic mice, with the aim of understanding how increased levels of SRF affect cardiac mitochondrial function. SRF is a central regulator of the expression of genes encoding structural proteins like actin and myosin, which are essential for maintaining cardiac function during increased workload [46, 47]. Age-related cardiovascular decline includes number of cardiac pathological processes such as hypertrophy and is often associated with diastolic dysfunction and many other complex changes. Increasing evidence links changes in SRF expression to various experimental and human diseases, suggesting its role in disease pathogenesis, with SRF expression notably increase in the aging heart [48–50]. This elevated SRF activity is associated with maladaptive hypertrophy, where prolonged or excessive hypertrophic signaling leads to cardiomyopathy, fibrosis, and ultimately heart failure [51].

Herein, we showed that SRF overexpression significantly reduced the lifespan of the transgenic mice, with the SRF-Tg mice surviving only until 6–7 months of age. At 6 months, these mice showed signs of cardiomyopathy, including increased heart size, weight, and chamber dilatation, which indicate progression of heart failure. Additionally, the heart-to-body weight ratio was significantly elevated, reflecting cardiac hypertrophy. Histological analysis revealed that SRF overexpression induces cardiomyocyte hypertrophy, as evidenced by larger cells and enlarged nuclei, and cardiac fibrosis, marked by interstitial fibrosis. The upregulation of BNP, a well-established marker of LV cardiac hypertrophy, cardiac stress and heart failure, further supports these findings, suggesting that SRF exacerbates pathological cardiac remodeling.

Prolonged cardiac overload inevitably drives heightened energy consumption, disrupting the delicate balance between mitochondrial energy production and the heart's metabolic demands [52]. Mitochondrial dysfunction arises early in compensatory cardiac hypertrophy, with these mitochondrial alterations potentially playing a pivotal role in the progression from compensatory hypertrophy to decompensated heart failure [53, 54]. Mitochondria and age-related disruptions in mitochondrial health are pivotal in the pathophysiology of cardiovascular aging. With aging, cardiovascular cells undergo mitochondrial dysfunction, marked by elevated mitochondrial-derived oxidative stress, accumulation of damaged mtDNA, compromised cellular energetics, and impaired mitochondrial quality control mechanisms [55–57]. Therefore, herein we examined the effects of SRF overexpression on mitochondrial function in the heart.

Transmission electron microscopy revealed significant mitochondrial damage, including shortened and fragmented cristae, along with a reduced mitochondrial DNA copy number, indicating impaired mitochondrial biogenesis and function. SRF overexpression also altered the balance between mitochondrial fission and fusion, evidenced by a reduction in the expression of PGC-1α and an increase in Drp1, a protein involved in mitochondrial fragmentation. These changes point to a disruption in mitochondrial dynamics that could impair cellular energy production, contributing to the observed heart dysfunction. Our findings are consistent with others that show the imbalance between mitochondrial fission and fusion ultimately contributes to the development of pathological cardiac hypertrophy [58].

Further analysis revealed that SRF overexpression disrupts oxidative phosphorylation, a key process for ATP production. The activity of electron transport chain complexes (I–IV) is significantly reduced, and the expression of key proteins involved in oxidative phosphorylation is downregulated. This impairment suggests that the heart cells may not generate sufficient ATP, which is essential for maintaining normal heart function.

Consistent with these findings, we transfected a human cardiomyocyte cell line (AC16) with an SRF plasmid. The results showed that SRF transfection in these cells reduced oxidative phosphorylation and increased glycolysis as a compensatory mechanism. This metabolic shift is similar to our observations in the mouse model, indicating that SRF overexpression impairs mitochondrial function and forces cells to rely more on glycolysis for ATP production. Additionally, SRF transfection led to a reduction in the expression of key mitochondrial regulators, such as PGC-1α and PGC-1β, and an increase in Drp1, further suggesting that SRF overexpression disrupts mitochondrial dynamics in both mouse and human cardiomyocytes.

Mitochondrial dysfunction and oxidative stress are intricately linked, which further exacerbates cellular injury and progression of pathological hypertrophy [59]. SRF overexpression induced oxidative stress, as shown by an increase in the biomarker 4-HNE, which indicates lipid peroxidation. This increase in oxidative stress is associated with a reduction in MnSOD, an important antioxidant enzyme, suggesting that the heart's antioxidant defense system is compromised. The accumulation of Ca^2+^ in the myoplasm, coupled with elevated oxidative stress, initiates a cascade of intracellular signaling pathways that drive cardiac hypertrophy [60, 61]. We showed that expression of SERCA2 and RyR2, which are crucial for calcium handling and myocardial contractility, was also reduced, suggesting that oxidative stress may impair calcium cycling and contribute to heart dysfunction.

The study further investigated the activation of the MAPK signaling pathway, which plays a critical role in cellular stress responses. Oxidative stress stimulates multiple MAPK signaling pathways [62, 63]. Furthermore, MAPKs through its complex network of signaling proteins, modulates gene expression and cellular remodeling processes, ultimately contributing to the pathogenesis of cardiac hypertrophy [64]. Dysregulation of this pathway can exacerbate hypertrophic progression and lead to heart failure [65]. We found persistent activation of p38 MAPK, JNK1/2, ERK1/2, and MEK in SRF-Tg mice, indicating chronic activation of stress-related kinases. This sustained activation suggests that SRF overexpression could trigger a prolonged stress response, contributing to the long-term pathological effects observed in these mice. Further, we found that the MAPK pathway is upregulated in wild-type mice as they age, highlighting its potential involvement in the cardiac aging process and age-related pathologies. This also suggests that SRF overexpression could be linked to age-related changes in cardiac signaling.

Recent studies have highlighted the SRF-cofilin-actin signaling pathway as a key regulator of mitochondrial, localization, and motility [34]. Additionally, our previous work emphasized SRF's role in regulating miR-21, a critical modulator of apoptosis that promotes cell survival and mitochondrial metabolism [66, 67]. It is therefore plausible that SRF exerts its effects through miR-21, which could influence mitochondrial function. Furthermore, our recent findings demonstrated mitochondrial dysfunction associated with the inhibition of the Rho/MRTF/SRF pathway [37]. Future studies will focus on measuring oxidative stress levels in SRF-knockdown cardiomyocytes and conducting RNA-seq experiments to further investigate the mechanisms underlying cardiac aging and mitochondrial dysfunction. In contrast to our SRF overexpressed transgenic mouse model, we have also generated a cardiac-specific antisense-mediated SRF transgenic mouse model with modestly reduced SRF levels, which has shown to delay the cardiac aging [68]. This suggests the therapeutic potential of using antisense-mediated reduction of SRF to delay cardiac aging, offering a potential novel strategy to combat age-related heart diseases.

In conclusion, this study provides compelling evidence that cardiac-specific overexpression of SRF induces a series of detrimental effects, including reduced lifespan, hypertrophy, fibrosis, mitochondrial dysfunction, oxidative stress, impaired calcium handling and activation of MAPKs. These changes likely contribute to the observed cardiomyopathy and heart failure. The results suggest that SRF plays a critical role in regulating cardiac remodeling and function, and its overexpression exacerbates pathological changes by disrupting mitochondrial function, shifting energy metabolism, and activating stress-related signaling pathways. Therefore, tightly regulating SRF expression in the heart is crucial in preventing the onset of cardiac diseases and mitigating mitochondrial dysfunction, emphasizing its potential as a therapeutic target for mitigating cardiac aging.

## Supplementary Information

Below is the link to the electronic supplementary material.

Supplementary file1 (DOCX 5858 KB)

Supplementary file2 (MP4 7289 KB)

## Data Availability

The raw data supporting the conclusions of this study will be made available by the corresponding author, without undue reservation.

## References

[1] Chen W, Zhao H, Li Y. Mitochondrial dynamics in health and disease: mechanisms and potential targets. Signal Transduct Target Ther. 2023;8(1):333. 10.1038/s41392-023-01547-9. 37669960 10.1038/s41392-023-01547-9 PMC10480456

[2] Atici AE, Crother TR, Noval Rivas M. Mitochondrial quality control in health and cardiovascular diseases. Front Cell Dev Biol. 2023;11:1290046. 10.3389/fcell.2023.1290046. 38020895 10.3389/fcell.2023.1290046 PMC10657886

[3] Tait SW, Green DR. Mitochondria and cell signalling. J Cell Sci. 2012;125(Pt 4):807–15. 10.1242/jcs.099234. 22448037 10.1242/jcs.099234 PMC3311926

[4] Finkel T, Menazza S, Holmström KM, Parks RJ, Liu J, Sun J, Liu J, Pan X, Murphy E. The ins and outs of mitochondrial calcium. Circ Res. 2015;116(11):1810–9. 10.1161/CIRCRESAHA.116.305484. 25999421 10.1161/CIRCRESAHA.116.305484 PMC6296495

[5] Waypa GB, Smith KA, Mungai PT, Dudley VJ, Helmin KA, Singer BD, Peek CB, Bass J, Nelson L, Shah SJ, Ofman G, Wasserstrom JA, Muller WA, Misharin AV, Budinger GRS, Abdala-Valencia H, Chandel NS, Dokic D, Bartom E, Zhang S, … Schumacker PT. Mitochondria regulate proliferation in adult cardiac myocytes. J Clin Investig. 2024;134(13):e165482. 10.1172/JCI165482. 10.1172/JCI165482 PMC11213516 38722697

[6] Nguyen TT, Wei S, Nguyen TH, Jo Y, Zhang Y, Park W, Gariani K, Oh CM, Kim HH, Ha KT, Park KS, Park R, Lee IK, Shong M, Houtkooper RH, Ryu D. Mitochondria-associated programmed cell death as a therapeutic target for age-related disease. Exp Mol Med. 2023;55(8):1595–619. 37612409 10.1038/s12276-023-01046-5 PMC10474116

[7] Ali MA, Gioscia-Ryan R, Yang D, Sutton NR, Tyrrell DJ. Cardiovascular aging: spotlight on mitochondria. Am J Physiol Heart Circ Physiol. 2024;326(2):H317–33. 10.1152/ajpheart.00632.202. 38038719 10.1152/ajpheart.00632.2023 PMC11219063

[8] Dai DF, Rabinovitch PS, Ungvari Z. Mitochondria and cardiovascular aging. Circ Res. 2012;110(8):1109–24. 10.1161/CIRCRESAHA.111.246140. 22499901 10.1161/CIRCRESAHA.111.246140 PMC3867977

[9] Sagar S, Gustafsson AB. Cardiovascular aging: the mitochondrial influence. J Cardiovasc Aging. 2023;3(3):33. 10.20517/jca.2023.22. 37583788 10.20517/jca.2023.22 PMC10426788

[10] Tocchi A, Quarles EK, Basisty N, Gitari L, Rabinovitch PS. Mitochondrial dysfunction in cardiac aging. Biochem Biophys Acta. 2015;1847(11):1424–33. 10.1016/j.bbabio.2015.07.009. 26191650 10.1016/j.bbabio.2015.07.009 PMC4575872

[11] Liang WJ, Gustafsson ÅB. The aging heart: mitophagy at the center of rejuvenation. Front Cardiovasc Med. 2020;7:18. 10.3389/fcvm.2020.00018. 32140472 10.3389/fcvm.2020.00018 PMC7042393

[12] Pagan LU, Gomes MJ, Gatto M, Mota GAF, Okoshi K, Okoshi MP. The role of oxidative stress in the aging heart. Antioxidants (Basel, Switzerland). 2022;11(2):336. 10.3390/antiox11020336. 35204217 10.3390/antiox11020336 PMC8868312

[13] Ranjbarvaziri S, Kooiker KB, Ellenberger M, Fajardo G, Zhao M, Vander Roest AS, Woldeyes RA, Koyano TT, Fong R, Ma N, Tian L, Traber GM, Chan F, Perrino J, Reddy S, Chiu W, Wu JC, Woo JY, Ruppel KM, Spudich JA, … Bernstein D. Altered cardiac energetics and mitochondrial dysfunction in hypertrophic cardiomyopathy. Circulation. 2021;144(21):1714–1731. 10.1161/CIRCULATIONAHA.121.053575 10.1161/CIRCULATIONAHA.121.053575 PMC8608736 34672721

[14] Matuz-Mares D, González-Andrade M, Araiza-Villanueva MG, Vilchis-Landeros MM, Vázquez-Meza H. Mitochondrial calcium: effects of its imbalance in disease. Antioxidants (Basel, Switzerland). 2022;11(5):801. 10.3390/antiox11050801. 35624667 10.3390/antiox11050801 PMC9138001

[15] Brown DA, Perry JB, Allen ME, Sabbah HN, Stauffer BL, Shaikh SR, Cleland JG, Colucci WS, Butler J, Voors AA, Anker SD, Pitt B, Pieske B, Filippatos G, Greene SJ, Gheorghiade M. Expert consensus document: Mitochondrial function as a therapeutic target in heart failure. Nat Rev Cardiol. 2017;14(4):238–50. 28004807 10.1038/nrcardio.2016.203 PMC5350035

[16] Quan Y, Xin Y, Tian G, Zhou J, Liu X. Mitochondrial ROS-modulated mtDNA: a potential target for cardiac aging. Oxidative Med Cell Longev. 2020;2020:9423593. 10.1155/2020/9423593. 10.1155/2020/9423593 PMC7139858 32308810

[17] Lin PH, Lee SH, Su CP, Wei YH. Oxidative damage to mitochondrial DNA in atrial muscle of patients with atrial fibrillation. Free Radical Biol Med. 2003;35(10):1310–8. 10.1016/j.freeradbiomed.2003.07.002. 14607530 10.1016/j.freeradbiomed.2003.07.002

[18] Lee MY, Park C, Ha SE, Park PJ, Berent RM, Jorgensen BG, Corrigan RD, Grainger N, Blair PJ, Slivano OJ, Miano JM, Ward SM, Smith TK, Sanders KM, Ro S. Serum response factor regulates smooth muscle contractility via myotonic dystrophy protein kinases and L-type calcium channels. PLoS ONE. 2017;12(2):e0171262. 10.1371/journal.pone.0171262. 28152551 10.1371/journal.pone.0171262 PMC5289827

[19] Deshpande A, Shetty PMV, Frey N, Rangrez AY. SRF: a seriously responsible factor in cardiac development and disease. J Biomed Sci. 2022;29(1):38. 10.1186/s12929-022-00820-3. 35681202 10.1186/s12929-022-00820-3 PMC9185982

[20] Wang D, Chang PS, Wang Z, Sutherland L, Richardson JA, Small E, Krieg PA, Olson EN. Activation of cardiac gene expression by myocardin, a transcriptional cofactor for serum response factor. Cell. 2001;105(7):851–62. 10.1016/s0092-8674(01)00404-4. 11439182 10.1016/s0092-8674(01)00404-4

[21] West AG, Shore P, Sharrocks AD. DNA binding by MADS-box transcription factors: a molecular mechanism for differential DNA bending. Mol Cell Biol. 1997;17(5):2876–87. 10.1128/MCB.17.5.2876. 9111360 10.1128/mcb.17.5.2876 PMC232140

[22] Strobeck M, Kim S, Zhang JC, Clendenin C, Du KL, Parmacek MS. Binding of serum response factor to CArG box sequences is necessary but not sufficient to restrict gene expression to arterial smooth muscle cells. J Biol Chem. 2001;276(19):16418–24. 10.1074/jbc.M100631200. 11279108 10.1074/jbc.M100631200

[23] Lu XG, Azhar G, Liu L, Tsou H, Wei JY. SRF binding to SRE in the rat heart: influence of age. J Gerontol Series A, Biol Sci Med Sci. 1998;53(1):B3–10. 10.1093/gerona/53a.1.b3. 10.1093/gerona/53a.1.b3 9467416

[24] Takahashi T, Schunkert H, Isoyama S, Wei JY, Nadal-Ginard B, Grossman W, Izumo S. Age-related differences in the expression of proto-oncogene and contractile protein genes in response to pressure overload in the rat myocardium. J Clin Investig. 1992;89(3):939–46. 10.1172/JCI115675. 1531837 10.1172/JCI115675 PMC442941

[25] Tsou H, Azhar G, Lu XG, Kovacs S, Peacocke M, Wei JY. Age-associated changes in basal c-fos transcription factor binding activity in rat hearts. Exp Cell Res. 1996;229(2):432–7. 10.1006/excr.1996.0388. 8986626 10.1006/excr.1996.0388

[26] Argentin S, Ardati A, Tremblay S, Lihrmann I, Robitaille L, Drouin J, Nemer M. Developmental stage-specific regulation of atrial natriuretic factor gene transcription in cardiac cells. Mol Cell Biol. 1994;14(1):777–90. 10.1128/mcb.14.1.777-790.1994. 8264645 10.1128/mcb.14.1.777-790.1994 PMC358426

[27] Parlakian A, Tuil D, Hamard G, Tavernier G, Hentzen D, Concordet JP, Paulin D, Li Z, Daegelen D. Targeted inactivation of serum response factor in the developing heart results in myocardial defects and embryonic lethality. Mol Cell Biol. 2004;24(12):5281–9. 10.1128/MCB.24.12.5281-5289.2004. 15169892 10.1128/MCB.24.12.5281-5289.2004 PMC419888

[28] Titus AS, V H, Kailasam S. Coordinated regulation of cell survival and cell cycle pathways by DDR2-dependent SRF transcription factor in cardiac fibroblasts. Am J Physiol Heart Circ Physiol. 2020;318(6):H1538–58. 10.1152/ajpheart.00740.2019. 32412792 10.1152/ajpheart.00740.2019

[29] Gineitis D, Treisman R. Differential usage of signal transduction pathways defines two types of serum response factor target gene. J Biol Chem. 2001;276(27):24531–9. 10.1074/jbc.M102678200. 11342553 10.1074/jbc.M102678200

[30] Zhao TC, Hines DS, Kukreja RC. Adenosine-induced late preconditioning in mouse hearts: role of p38 MAP kinase and mitochondrial K(ATP) channels. Am J Physiol Heart Circ Physiol. 2001;280(3):H1278–85. 10.1152/ajpheart.2001.280.3.H1278. 11179074 10.1152/ajpheart.2001.280.3.H1278

[31] Kong JY, Klassen SS, Rabkin SW. Ceramide activates a mitochondrial p38 mitogen-activated protein kinase: a potential mechanism for loss of mitochondrial transmembrane potential and apoptosis. Mol Cell Biochem. 2005;278(1–2):39–51. 10.1007/s11010-005-1979-6. 16180087 10.1007/s11010-005-1979-6

[32] Zhang X, Azhar G, Chai J, Sheridan P, Nagano K, Brown T, Yang J, Khrapko K, Borras AM, Lawitts J, Misra RP, Wei JY. Cardiomyopathy in transgenic mice with cardiac-specific overexpression of serum response factor. Am J Physiol Heart Circ Physiol. 2001;280(4):H1782–92. 10.1152/ajpheart.2001.280.4.H1782. 11247792 10.1152/ajpheart.2001.280.4.H1782

[33] Zhang X, Azhar G, Furr MC, Zhong Y, Wei JY. Model of functional cardiac aging: young adult mice with mild overexpression of serum response factor. Am J Physiol Regul Integr Comp Physiol. 2003;285(3):R552–60. 10.1152/ajpregu.00631.2002. 12909581 10.1152/ajpregu.00631.2002

[34] Moore ML, Wang GL, Belaguli NS, Schwartz RJ, McMillin JB. GATA-4 and serum response factor regulate transcription of the muscle-specific carnitine palmitoyltransferase I beta in rat heart. J Biol Chem. 2001;276(2):1026–33. 10.1074/jbc.M009352200. 11038368 10.1074/jbc.M009352200

[35] Beck H, Flynn K, Lindenberg KS, Schwarz H, Bradke F, Di Giovanni S, Knöll B. Serum Response Factor (SRF)-cofilin-actin signaling axis modulates mitochondrial dynamics. Proc Natl Acad Sci USA. 2012;109(38):E2523–32. 10.1073/pnas.1208141109. 22927399 10.1073/pnas.1208141109 PMC3458318

[36] Patyal P, Nguyen B, Zhang X, Azhar G, Ameer FS, Verma A, Crane J, Kc G, Che Y, Wei JY. Rho/SRF inhibitor modulates mitochondrial functions. Int J Mol Sci. 2022;23(19):11536. 10.3390/ijms231911536. 36232837 10.3390/ijms231911536 PMC9570101

[37] Patyal P, Zhang X, Verma A, Azhar G, Wei JY. Inhibitors of Rho/MRTF/SRF transcription pathway regulate mitochondrial function. Cells. 2024;13(5):392. 10.3390/cells13050392. 38474356 10.3390/cells13050392 PMC10931493

[38] Kilkenny C, Browne WJ, Cuthill IC, Emerson M, Altman DG. Improving bioscience research reporting: the ARRIVE guidelines for reporting animal research. PLoS Biol. 2010;8(6):e1000412. 10.1371/journal.pbio.1000412. 20613859 10.1371/journal.pbio.1000412 PMC2893951

[39] Azhar G, Nagano K, Patyal P, Zhang X, Verma A, Wei JY. Deletion of interleukin-1β converting enzyme alters mouse cardiac structure and function. Biology. 2024;13(3):172. 10.3390/biology13030172. 38534442 10.3390/biology13030172 PMC10968068

[40] Verma A, Azhar G, Patyal P, Zhang W, Zhang X, Wei JY. Proteomic analysis of P. gingivalis-Lipopolysaccharide induced neuroinflammation in SH-SY5Y and HMC3 cells. GeroScience. 2024;46(5):4315–32. 10.1007/s11357-024-01117-z. 38507186 10.1007/s11357-024-01117-z PMC11336124

[41] Zhang X, Azhar G, Zhong Y, Wei JY. Identification of a novel serum response factor cofactor in cardiac gene regulation. J Biol Chem. 2004;279(53):55626–32. 10.1074/jbc.M405945200. 15492011 10.1074/jbc.M405945200

[42] Patyal P, Ameer FS, Verma A, Zhang X, Azhar G, Shrivastava J, Sharma S, Zhang R, Wei JY. The role of sirtuin-1 isoforms in regulating mitochondrial function. Curr Issues Mol Biol. 2024;46(8):8835–51. 10.3390/cimb46080522. 39194739 10.3390/cimb46080522 PMC11352618

[43] Verma A, Azhar G, Zhang X, Patyal P, Kc G, Sharma S, Che Y, Wei JY. P. gingivalis-LPS induces mitochondrial dysfunction mediated by neuroinflammation through oxidative stress. Int J Mol Sci. 2023;24(2):950. 10.3390/ijms24020950. 36674463 10.3390/ijms24020950 PMC9861869

[44] Sharma S, Zhang X, Azhar G, Patyal P, Verma A, Kc G, Wei JY. Valine improves mitochondrial function and protects against oxidative stress. Biosci Biotechnol Biochem. 2024;88(2):168–76. 10.1093/bbb/zbad169. 38093456 10.1093/bbb/zbad169 PMC10807754

[45] Nolfi-Donegan D, Braganza A, Shiva S. Mitochondrial electron transport chain: oxidative phosphorylation, oxidant production, and methods of measurement. Redox Biol. 2020;37:101674. 10.1016/j.redox.2020.101674. 32811789 10.1016/j.redox.2020.101674 PMC7767752

[46] Miano JM, Long X, Fujiwara K. Serum response factor: master regulator of the actin cytoskeleton and contractile apparatus. Am J Physiol Cell Physiol. 2007;292(1):C70–81. 10.1152/ajpcell.00386.2006. 16928770 10.1152/ajpcell.00386.2006

[47] Schratt G, Philippar U, Berger J, Schwarz H, Heidenreich O, Nordheim A. Serum response factor is crucial for actin cytoskeletal organization and focal adhesion assembly in embryonic stem cells. J Cell Biol. 2002;156(4):737–50. 10.1083/jcb.200106008. 11839767 10.1083/jcb.200106008 PMC2174087

[48] Miano JM. Role of serum response factor in the pathogenesis of disease. Lab Inv J Tech Methods Pathol. 2010;90(9):1274–84. 10.1038/labinvest.2010.104. 10.1038/labinvest.2010.104 20498652

[49] Mengmeng X, Yuejuan X, Sun C, Yanan L, Fen L, Kun S. Novel mutations of the SRF gene in Chinese sporadic conotruncal heart defect patients. BMC Med Genet. 2020;21(1):95. 10.1186/s12881-020-01032-y. 32380971 10.1186/s12881-020-01032-y PMC7203814

[50] Xiao S, Liang R, Muili AB, Cao X, Navran S, Schwartz RJ, Iyer D. Mutant SRF and YAP synthetic modified mRNAs drive cardiomyocyte nuclear replication. J Cardiovasc Aging. 2022;2:29. 10.20517/jca.2022.17. 10.20517/jca.2022.20 PMC9311335 35891703

[51] Nelson TJ, Balza R Jr, Xiao Q, Misra RP. SRF-dependent gene expression in isolated cardiomyocytes: regulation of genes involved in cardiac hypertrophy. J Mol Cell Cardiol. 2005;39(3):479–89. 10.1016/j.yjmcc.2005.05.004. 15950986 10.1016/j.yjmcc.2005.05.004

[52] Werbner B, Tavakoli-Rouzbehani OM, Fatahian AN, Boudina S. The dynamic interplay between cardiac mitochondrial health and myocardial structural remodeling in metabolic heart disease, aging, and heart failure. J Cardiovasc Aging. 2023;3(1):9. 10.20517/jca.2022.42. 36742465 10.20517/jca.2022.42 PMC9894375

[53] Müller OJ, Heckmann MB, Ding L, Rapti K, Rangrez AY, Gerken T, Christiansen N, Rennefahrt UEE, Witt H, González Maldonado S, Ternes P, Schwab DM, Ruf T, Hille S, Remes A, Jungmann A, Weis TM, Kreußer JS, Gröne HJ, Backs J, … Frey N. Comprehensive plasma and tissue profiling reveals systemic metabolic alterations in cardiac hypertrophy and failure. Cardiovasc Res. 2019;115(8):1296–1305. 10.1093/cvr/cvy274 10.1093/cvr/cvy274 30418544

[54] Dai DF, Johnson SC, Villarin JJ, Chin MT, Nieves-Cintrón M, Chen T, Marcinek DJ, Dorn GW 2nd, Kang YJ, Prolla TA, Santana LF, Rabinovitch PS. Mitochondrial oxidative stress mediates angiotensin II-induced cardiac hypertrophy and Galphaq overexpression-induced heart failure. Circ Res. 2011;108(7):837–46. 10.1161/CIRCRESAHA.110.232306. 21311045 10.1161/CIRCRESAHA.110.232306 PMC3785241

[55] Song M, Franco A, Fleischer JA, Zhang L, Dorn GW 2nd. Abrogating mitochondrial dynamics in mouse hearts accelerates mitochondrial senescence. Cell Metab. 2017;26(6):872-883.e5. 10.1016/j.cmet.2017.09.023. 29107503 10.1016/j.cmet.2017.09.023 PMC5718956

[56] Torregrosa-Muñumer R, Forslund JME, Goffart S, Pfeiffer A, Stojkovič G, Carvalho G, Al-Furoukh N, Blanco L, Wanrooij S, Pohjoismäki JLO. PrimPol is required for replication reinitiation after mtDNA damage. Proc Natl Acad Sci USA. 2017;114(43):11398–403. 10.1073/pnas.1705367114. 29073063 10.1073/pnas.1705367114 PMC5664498

[57] Giorgi C, Marchi S, Simoes ICM, Ren Z, Morciano G, Perrone M, Patalas-Krawczyk P, Borchard S, Jędrak P, Pierzynowska K, Szymański J, Wang DQ, Portincasa P, Węgrzyn G, Zischka H, Dobrzyn P, Bonora M, Duszynski J, Rimessi A, Karkucinska-Wieckowska A, … Wieckowski MR. Mitochondria and reactive oxygen species in aging and age-related diseases. Int Rev Cell Mol Biol. 2018;340:209–344. 10.1016/bs.ircmb.2018.05.006 10.1016/bs.ircmb.2018.05.006 PMC8127332 30072092

[58] Liu X, Guo C, Zhang Q. Novel insights into the involvement of mitochondrial fission/fusion in heart failure: from molecular mechanisms to targeted therapies. Cell Stress Chaperones. 2023;28(2):133–44. 10.1007/s12192-023-01321-4. 36652120 10.1007/s12192-023-01321-4 PMC10050249

[59] Peoples JN, Saraf A, Ghazal N, Pham TT, Kwong JQ. Mitochondrial dysfunction and oxidative stress in heart disease. Exp Mol Med. 2019;51(12):1–13. 10.1038/s12276-019-0355-7. 31857574 10.1038/s12276-019-0355-7 PMC6923355

[60] Michelucci A, Liang C, Protasi F, Dirksen RT. Altered Ca2+ handling and oxidative stress underlie mitochondrial damage and skeletal muscle dysfunction in aging and disease. Metabolites. 2021;11(7):424. 10.3390/metabo11070424. 34203260 10.3390/metabo11070424 PMC8304741

[61] Dridi H, Santulli G, Bahlouli L, Miotto MC, Weninger G, Marks AR. Mitochondrial calcium overload plays a causal role in oxidative stress in the failing heart. Biomolecules. 2023;13(9):1409. 10.3390/biom13091409. 37759809 10.3390/biom13091409 PMC10527470

[62] Son Y, Cheong YK, Kim NH, Chung HT, Kang DG, Pae HO. Mitogen-activated protein kinases and reactive oxygen species: how can ROS activate MAPK pathways? J Signal Transduct. 2011;2011:792639. 10.1155/2011/792639. 21637379 10.1155/2011/792639 PMC3100083

[63] Rezatabar S, Karimian A, Rameshknia V, Parsian H, Majidinia M, Kopi TA, Bishayee A, Sadeghinia A, Yousefi M, Monirialamdari M, Yousefi B. RAS/MAPK signaling functions in oxidative stress, DNA damage response and cancer progression. J Cell Physiol. 2019;234(9):14951–65. 10.1002/jcp.28334. 30811039 10.1002/jcp.28334

[64] Zhang W, Elimban V, Nijjar MS, Gupta SK, Dhalla NS. Role of mitogen-activated protein kinase in cardiac hypertrophy and heart failure. Exp Clin Cardiol. 2003;8(4):173–83. 19649217 PMC2719157

[65] Streicher JM, Ren S, Herschman H, Wang Y. MAPK-activated protein kinase-2 in cardiac hypertrophy and cyclooxygenase-2 regulation in heart. Circ Res. 2010;106(8):1434–43. 10.1161/CIRCRESAHA.109.213199. 20339119 10.1161/CIRCRESAHA.109.213199 PMC2903446

[66] Zhang X, Azhar G, Helms SA, Wei JY. Regulation of cardiac microRNAs by serum response factor. J Biomed Sci. 2011;18(1):15. 10.1186/1423-0127-18-15. 21303526 10.1186/1423-0127-18-15 PMC3048499

[67] Sikora M, Śmieszek A, Pielok A, Marycz K. MiR-21-5p regulates the dynamic of mitochondria network and rejuvenates the senile phenotype of bone marrow stromal cells (BMSCs) isolated from osteoporotic SAM/P6 mice. Stem Cell Res Ther. 2023;14(1):54. 10.1186/s13287-023-03271-1. 36978118 10.1186/s13287-023-03271-1 PMC10053106

[68] Azhar G, Zhang X, Wang S, Zhong Y, Quick CM, Wei JY. Maintaining serum response factor activity in the older heart equal to that of the young adult is associated with better cardiac response to isoproterenol stress. Basic Res Cardiol. 2007;102(3):233–44. 10.1007/s00395-006-0634-z. 17122890 10.1007/s00395-006-0634-z

