# Supplementary material for: Cardiac-specific overexpression of serum response factor regulates age-associated decline in mitochondrial function
Source: GeroScience. 2025 Mar 31;47(5):6565–82. doi: 10.1007/s11357-025-01629-2 (PMC12634980; doi:10.1007/s11357-025-01629-2)
Supplement: Supplementary file 1 — Supplementary file1 (DOCX 5858 KB) [file 11357_2025_1629_MOESM1_ESM.docx]

Title: Cardiac-specific overexpression of serum response factor regulates age-associated decline in mitochondrial function

Authors: Pankaj Patyal, Gohar Azhar, Xiaomin Zhang, Ambika Verma, Jeanne Y. Wei^*^

**Affiliation:**
Donald W. Reynolds Department of Geriatrics and Institute on Aging, University of Arkansas for Medical Sciences, Little Rock, AR 72205, USA

Figure Legend Supplementary Fig. 1

Video recording of SRF-Tg and non-Tg littermate. Video recording demonstrates the phenotype of SRF-Tg mouse. At 6 months of age, during the near-death stage, this mouse exhibited very rapid breathing and a limp body with minimal to no reflex responses, in contrast to its non-Tg littermate.

Supplementary Fig. 2


 A)

B)

**Segmented images of H & E staining from Fig. 2** (**A**) Left ventricular cardiac myocytes of SRF-Tg and non-Tg mice at 6 months of age stained with hematoxylin and eosin stain. (**B**) Segmented images with marked edges of H&E staining for non-Tg and SRF-Tg mouse heart sections from Fig. A, generated through ImageJ software.

Supplementary Fig. 3

**Western blot analysis of SRF at 2 months of age.** (**A**) Representative western blot images of SRF, in SRF-Tg and non-Tg mouse hearts at 2 months of age. GAPDH was used as loading control. (**B**) Relative quantification of protein levels of SRF normalized against GAPDH at 2 months of age, shows no change in the protein expression in SRF-Tg mice hearts as compared to non-Tg littermates. Data are mean ± SD of 5 repeats. ns ≥ .05, using one-way ANOVA with Tukey’s procedure.
